# Osteoblastoma of the Mandible: A Case Report

**DOI:** 10.7759/cureus.68629

**Published:** 2024-09-04

**Authors:** Samyuktha Aarthi, Karthikeyan Ramalingam, Pratibha Ramani, Pradeep D

**Affiliations:** 1 Oral Pathology and Microbiology, Saveetha Dental College and Hospitals, Saveetha Institute of Medical and Technical Sciences, Saveetha University, Chennai, IND; 2 Oral and Maxillofacial Surgery, Saveetha Dental College and Hospitals, Saveetha Institute of Medical and Technical Sciences, Saveetha University, Chennai, IND

**Keywords:** bone tumors, external root resorption, biopsy, bone expansion, root resorption, mandibular molar region, mandible, jaw bones, jaws, osteoblastoma

## Abstract

Osteoblastoma is a rare, benign bone tumor primarily affecting the spine but can occasionally involve the jaws, particularly the mandible. This report discusses the case of a 38-year-old female patient presenting with mild pain and swelling in the left mandibular region for the past few months. Clinical, radiographic, and histopathological evaluation confirmed the diagnosis of osteoblastoma. A conservative surgical excision was performed. No recurrence was observed on follow-up. Differential diagnoses included low-grade osteosarcoma, osteoid osteoma, and other bone-forming lesions. This case report emphasizes the importance of distinguishing osteoblastoma from similar lesions to ensure appropriate treatment and favorable prognosis.

## Introduction

Non-odontogenic tumors of jaws encompass many entities. However, frequently encountered pathologies comprise osteosarcoma, fibrous dysplasia, central giant cell granuloma, osteoma, osteochondroma, and osteoblastoma (OB) [1,2]. OB is a rare benign neoplasm that primarily affects the spine but can also be seen in the jaw bones, especially in the mandible. It can present as an aggressive variant with local destruction and high recurrence similar to a low-grade osteosarcoma [2,3]. OB is an uncommon, noncancerous bone tumor recognized as a distinct entity that should be distinguished from osteoid osteoma. Although generally regarded as benign, the exact nature of OB remains unclear. Jaffe and Lichtenstein identified OB as a true tumor arising from osteoblasts. However, other pathogenetic concepts include abnormal local response to trauma or injury or localized physiological changes in bone as a cause for OB [3,4].

PUBMED search with MESH keywords ("osteoblastoma"[All Fields]) AND ("jaws"[All Fields]) revealed only 31 results for cases of OB involving the jawbones [5]. Only a limited number of OB cases have been reported in the literature and the jaw bone involvement is infrequent. OB is categorized into three types: periosteal, medullary, and cortical. While cortical OB is commonly found in extragnathic locations, jaw OB is usually medullary or periosteal. The mandible is more frequently affected than the maxilla. Of all OB cases, 41% occur in women and 59% in men [3-5]. Radiographs are crucial in identifying hard tissue lesions, especially in the maxillofacial region. OB and osteoid osteoma are two distinct entities according to the World Health Organization (WHO) classification but both entities are rare in the maxilla and mandible. OB can expand and erode the surrounding bone [6,7]. OB is usually managed by interdisciplinary collaboration with surgeons, radiologists, and pathologists. Recurrence is seen in 10% to 20% of patients [2].

This case report discusses a rare entity of OB involving the mandible of a middle-aged female in the posterior region. We have also compared OB with differential diagnoses of similar bone pathologies.

## Case presentation

A 38-year-old female patient presented to the outpatient department of Saveetha Dental College and Hospitals, Chennai, India. Her chief complaint was mild pain and swelling in the left lower back tooth region for the past four months. The patient was asymptomatic for five months. She noticed a swelling on the lower border of the left mandible a few weeks ago. She began experiencing mild pain, which prompted her visit to the dentist. The pain was localized to the lower jaw, spontaneous in nature, continuous, and dull-aching type with gradually increasing intensity. It was aggravated by mastication and relieved by analgesics. Past medical history, past surgical history, and past dental history were non-contributory. Informed consent was obtained from the patient for further investigations and surgical treatment. 

**Figure 1:**
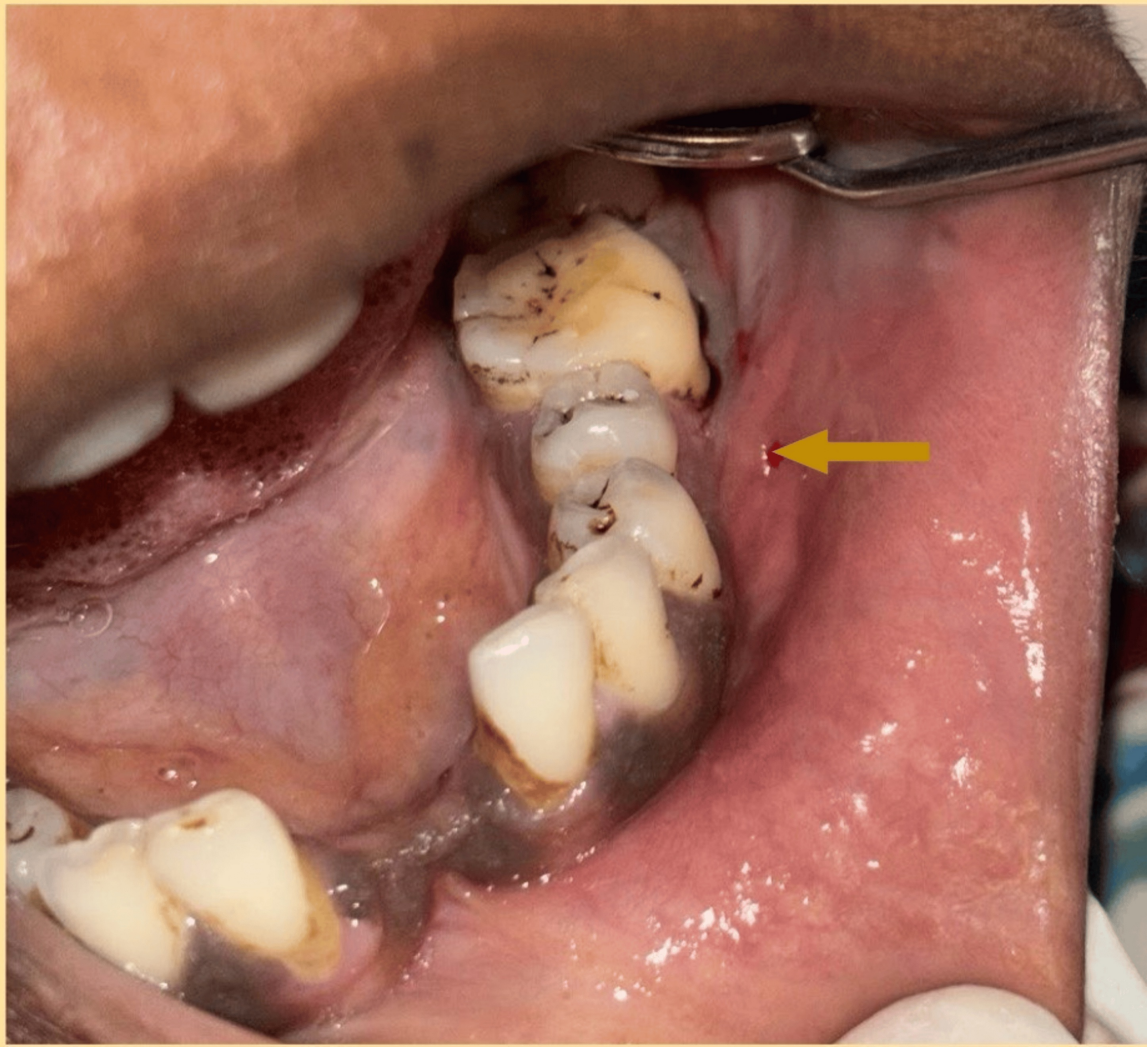
Intraoral clinical picture showing obliteration of the buccal vestibule in the mandibular region

On intraoral examination, there was an oval solitary swelling extending from 34 to 37 region with obliteration of buccal vestibule (Figure 1). It was measuring 3.2x2.3x1.5 cm in size. The surface appeared normal, with no visible signs of inflammation. The swelling was tender on palpation, with a well-defined border and a bony hard consistency. The buccal expansion was palpable. It was not compressible, did not have palpable pulsations, and there was no mobility. There was no increase in surface temperature. Lymph nodes were not palpable, and no other significant intraoral findings were observed.

**Figure 2:**
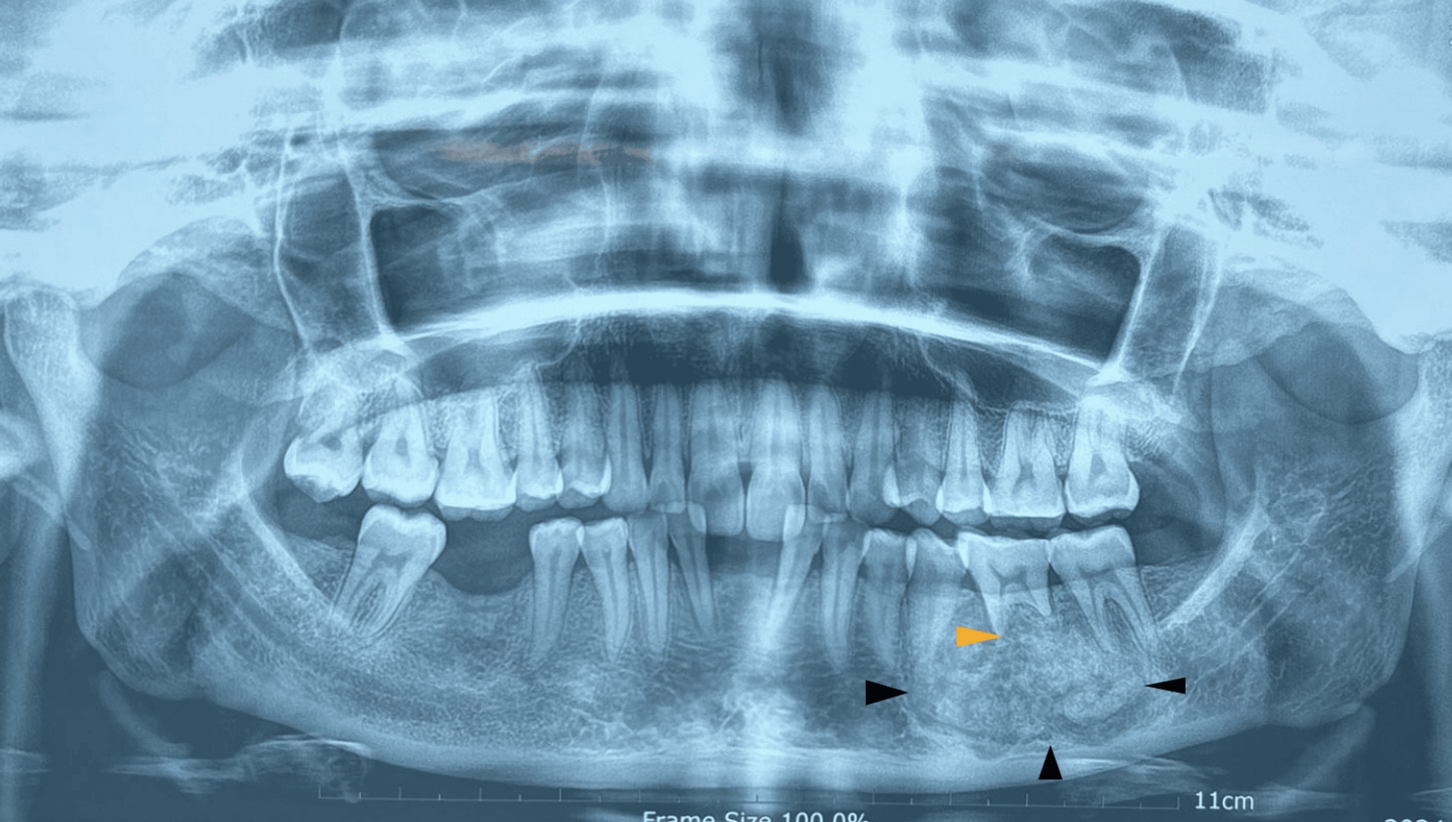
Orthopantomogram showing well-defined mixed radiopaque and radiolucent mass as shown by the black arrowhead, with root resorption of mandibular molar as shown by the yellow arrowhead.

The orthopantomogram revealed a well-defined, mixed radiopaque-radiolucent lesion from the distal aspect of the root of 34 extending to the medial aspect of 37, with root resorption of mesial and distal roots of 36 (Figure 2).

**Figure 3:**
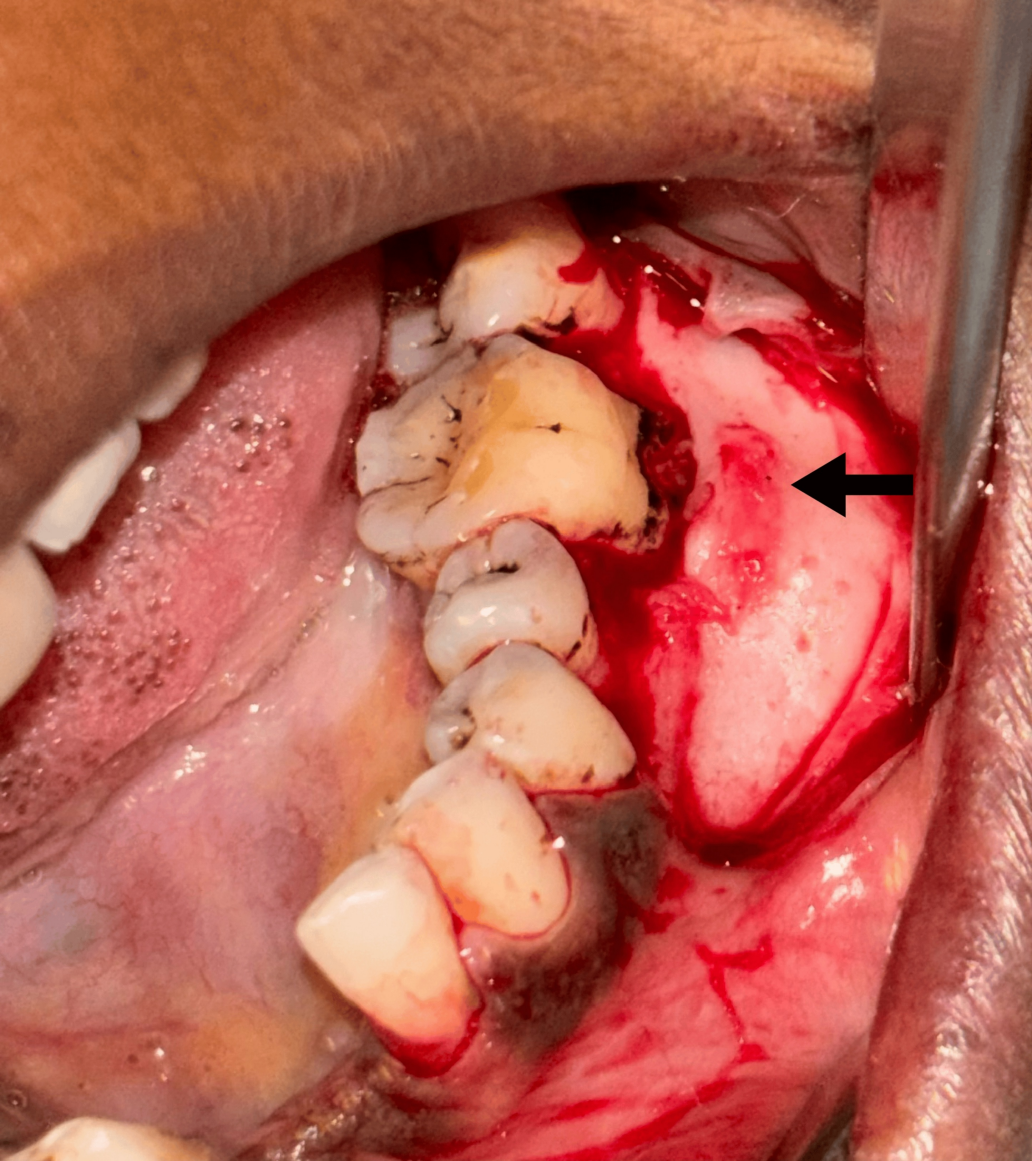
Surgical exposure of the bony lesion on buccal aspect of the mandible The arrow depicts the expanded buccal cortical plate.

Under local anesthesia, a mucoperiosteal flap was reflected and it showed obvious buccal cortical plate expansion (Figure 3). An incisional biopsy was taken from the representative site and the flap was closed by silk sutures. The incisional biopsy sample was sent to the Department of Oral Pathology for further processing.

**Figure 4:**
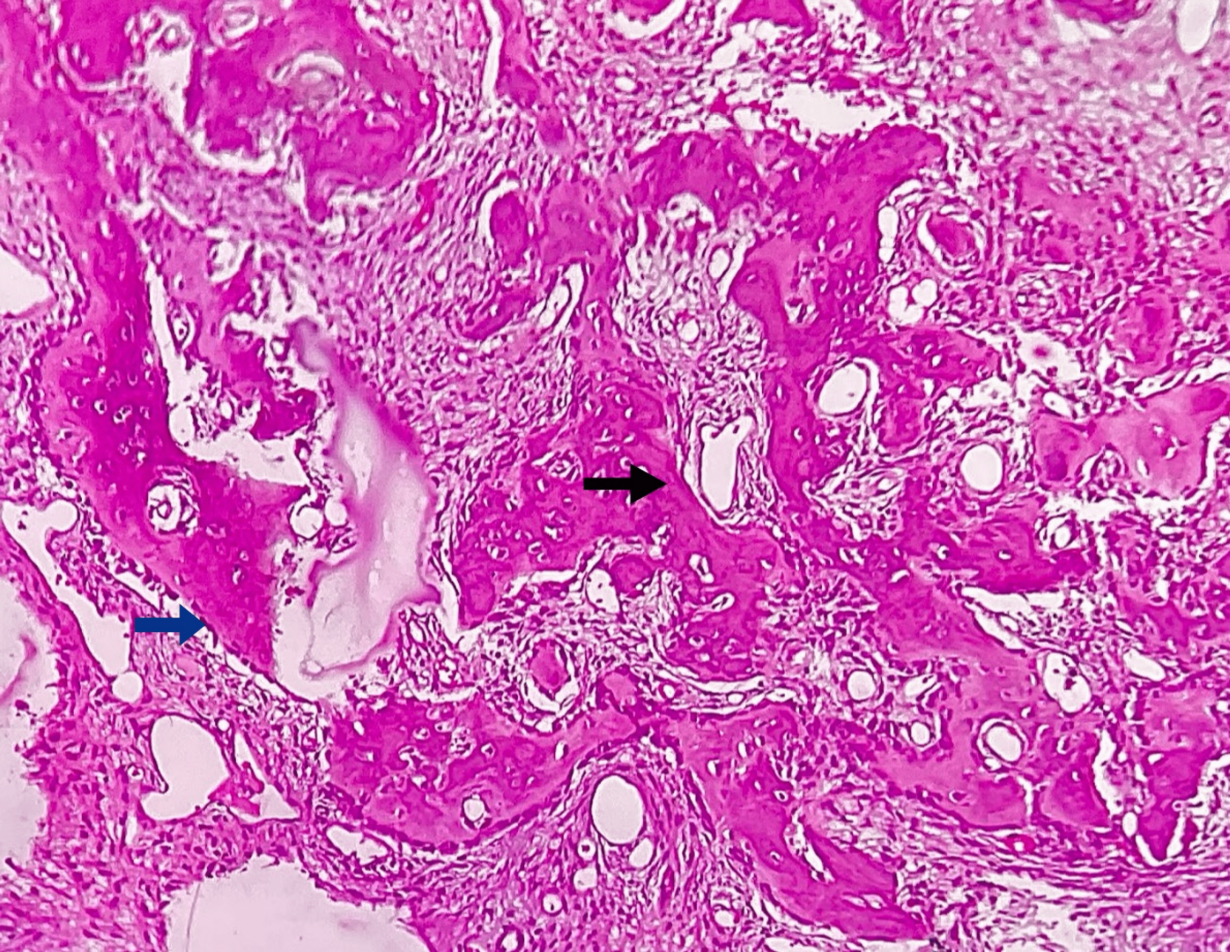
Photomicrograph of incisional biopsy Histopathological features show irregular bony trabeculae with peri-trabecular clefting (black arrow), osteocytes in lacunae and peripheral osteoblastic rimming (blue arrow), and rich, vascular connective tissue stroma.

The histopathological features revealed that H&E-stained sections show numerous irregular bony trabeculae of variable size and shape, with areas of osteoid surrounded by dense sclerotic bone at the periphery. The bony trabeculae showed osteoblastic rimming along with prominent resting and reversal lines, lacunae with osteocytes, and peri-trabecular clefting. A few multinucleated giant cells were also noted, suggesting osteoclastic giant cells. The intervening fibrovascular connective tissue shows rich vascularity with numerous dilated blood vessels, inflammatory cells, and areas of hemorrhage. Correlating clinical and radiographical features, histopathology was suggestive of OB (Figure 4).

**Figure 5:**
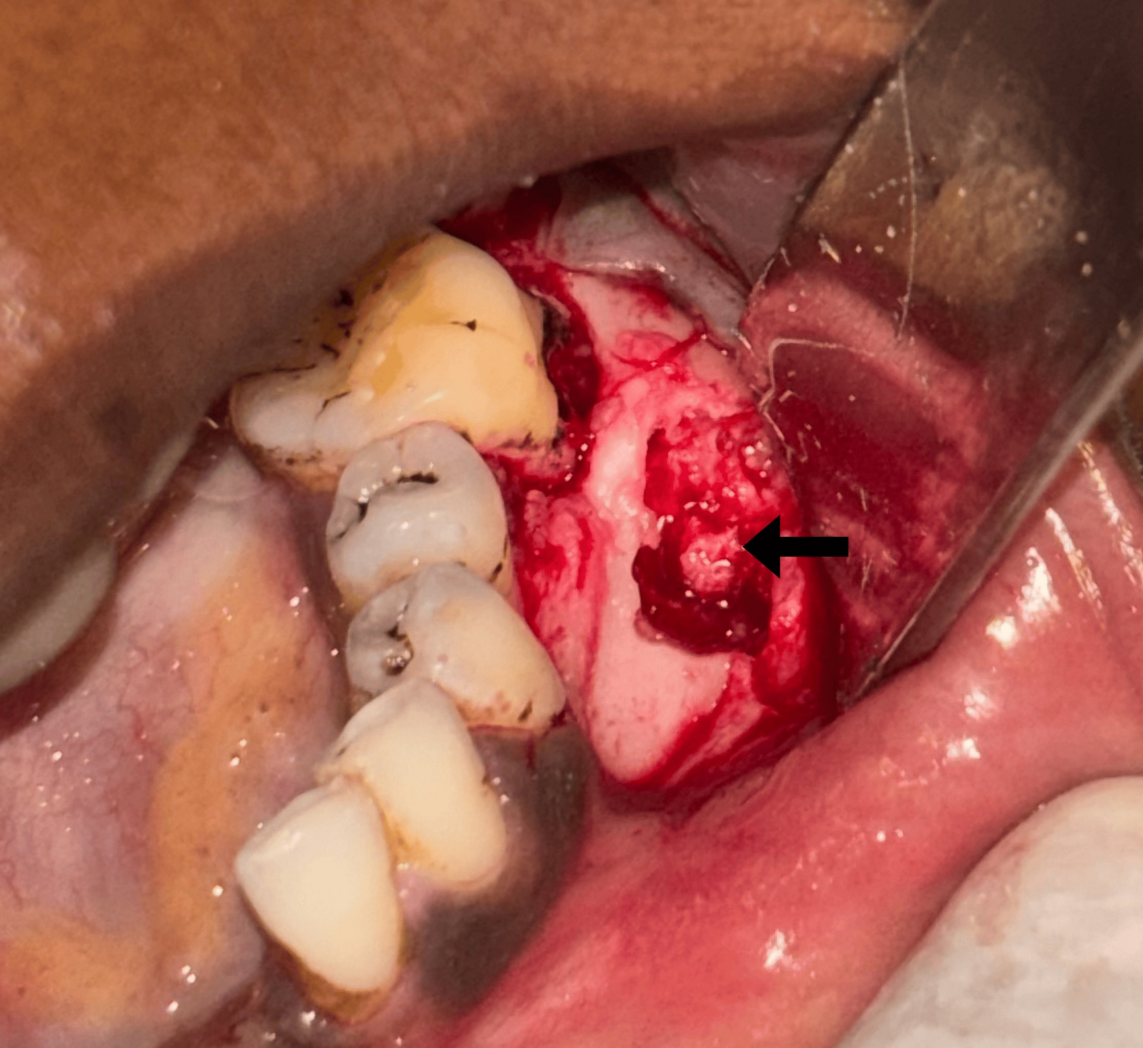
Intraoperative picture showing the biopsy procedure from the mandible

Based on the histopathological diagnosis, conservative surgical curettage with preservation of buccal and lingual cortices was performed under general anesthesia (Figure 5).

**Figure 6:**
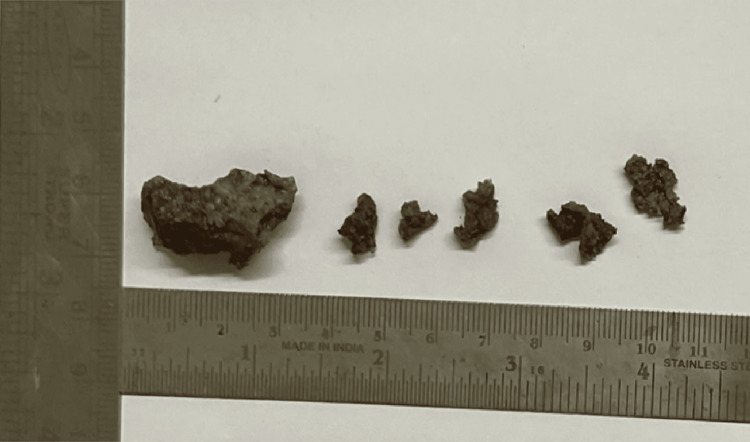
Excisional biopsy specimens

The excisional biopsy sample was sent to the Oral Pathology Department for further processing. Multiple bits of hard-tissue specimen were received in formalin. They were brownish in color and hard in consistency, and the largest bit measured 2.9x1.4x1.3 cm in size (Figure 6). All the hard-tissue bits were kept for decalcification in 10% formic acid for four days and then subjected to routine tissue processing.

**Figure 7:**
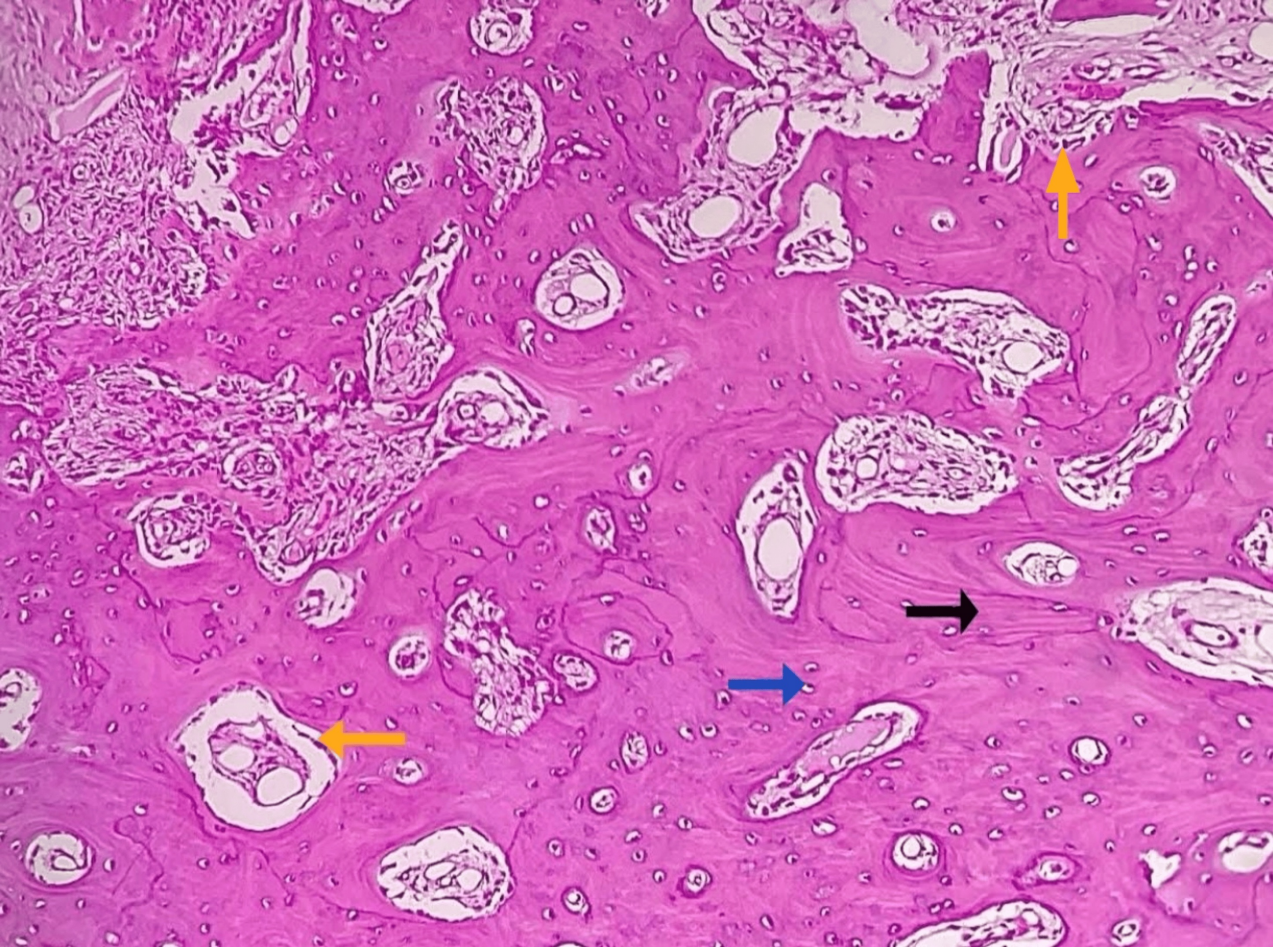
Photomicrograph of excisional biopsy The figure shows irregular bony trabeculae with osteoblastic rimming (yellow arrow), numerous resting lines (black arrow) and reversal lines, osteocytes within lacunae (blue arrow), and connective tissue stroma with vascular spaces (H&E, 20x).

On histopathological examination, H&E-stained decalcified section revealed numerous irregular bony trabeculae of variable size and shape along with many areas of osteoid in a fibrovascular connective tissue stroma showing rich vascularity with numerous dilated and congested blood vessels with extravasated RBCs. The bony trabeculae showed few areas of osteoblastic rimming along with resting and reversal lines. Lacunae containing osteocytes were seen (Figure 7). Multinucleated giant cells suggestive of osteoclasts, few acellular basophilic calcifications, and chronic inflammatory cells predominantly lymphocytes were also noted. These findings were suggestive of OB. 

The patient remains disease-free on follow-up and is under regular monitoring.

## Discussion

OB is a benign, slow-growing lesion comprising only 1% of bone tumors and 1-5% of benign tumors [5]. It is marked by the production of osteoid and bone, along with a high density of osteoblasts. It usually affects men with a mean age of 23 years. Seventy-four percent of OB cases present with mandibular involvement [6]. Our patient was a 38-year-old female with a lesion in the mandibular molar region. Plain radiographs, computed tomography scans, magnetic resonance imaging, and nuclear medicine imaging have been used to determine the extent and aggressiveness of OB [5]. Our patient presented with a well-defined, mixed radiopaque-radiolucent lesion in the mandibular molar region.

Differential diagnoses of OB based on radiographic features include ossifying fibroma, OB, osteosarcoma, osteoid osteoma, focal cemento-osseous dysplasia, and calcifying odontogenic cyst with odontoma [3-5]. An osteosarcoma typically features ill-defined margins and exhibits a substantial area of sclerotic bone formation, which is atypical for an ossifying fibroma. Focal cemento-osseous dysplasia is unlikely due to the expansive nature of our case [6]. Osteoid osteoma is clinically characterized by its smaller size (less than 2 cm), nocturnal pain exacerbation, and positive response to salicylates and non-steroidal anti-inflammatory drugs. Cementoblastoma typically occurs as a rounded radiopacity located at the apical half of a single-rooted tooth, and is associated with odontogenic pain. Ossifying fibroma presents with a spherical-shaped mixed radiolucency and is generally painless [7,8]. 

Moshref et al. have reported an uncommon instance of multifocal OB in a woman aged 30, affecting the maxilla and the mandible. The patient had surgery to remove the tumor, reconstruction using an iliac bone graft, and implants. Total surgical excision is required for a good prognosis of OB. The patient was monitored for four years following the procedure, and neither the panoramic view nor the clinical examination revealed any indications of recurrence [9]. The literature states that OB might manifest symptomatically or be unintentionally found during a regular checkup. It can produce facial asymmetry by the increase in tissue volume. In the OB cases, 7.2% to 50% are asymptomatic [10-12]. Our patient presented with a dull pain associated with swelling in the mandibular region.

On radiography, OB exhibits a range of traits and patterns. These lesions may appear as sclerotic masses, lytic lesions, or mixed pictures on radiography. The tumor's size and calcification intensity determine the radiographic characteristics. It is characterized by clearly defined edges and the enlargement of the cortical bones. Krishnan et al. [10] reported cone beam computed tomography (CBCT) findings of an osteoblastoma involving the mandible as a radiopaque, non-homogeneous mass without root resorption. Osteoblastomas typically present as poorly defined, radiolucent/radiopaque lesions with calcifications without periosteal response or sclerotic boundary [12-16]. A retrospective analysis was conducted by Capodiferro et al. [14] on four benign osteoblastoma with clinical, radiological, and histopathologic characteristics of each case. Their case series had young patients. They were two males and two females, with ages ranging from 10 to 21 years presenting with excruciating bone growth in their posterior mandible.

Formation of osteoid and immature woven bony trabeculae with osteoblastic rimming are important features of OB. They also present areas of extravasated erythrocytes in a vascularized fibrous connective tissue stroma [14-16]. Kaur et al. [17] have stressed the importance of differential diagnosis among various aggressive bony lesions involving the mandible. The clinical and radiological findings, gross pathology, and histopathological features were consistent with the reported literature. OB is frequently confused with osteoid osteoma. As discussed earlier, a larger lesion without pain or bony sclerosis is a diagnostic feature [18]. Lack of peripheral permeation or infiltration, nuclear beta-catenin staining, and positivity to microRNA-210 differentiate it from osteosarcoma [5]. SATB2 can be useful in histologic samples with low bone production to identify cells with osteoblast differentiation and distinguish neoplastic bone caused by tumor mesenchymal cells from periosteal bone reaction [19].

Taghsimi et al. have reported that dental implantation was effective in 84.2% of instances that were described, i.e. in the cases of osteoid osteoma, odontoma, cementoblastoma, idiopathic osteosclerosis, and condensing osteitis. Dental implants were not widely used in OB and cemento-osseous dysplasia due to the risk of lesion recurrence and implant failure [20]. The primary treatment option for OB is surgery with curettage or en-bloc resection based on the clinical and radiological findings [5]. The Iliac bone crest is preferred for reconstruction. It is usually the gold standard for autogenous bone grafts due to its brilliant osteogenetic, osteoconductive, and osteoinductive properties [21]. Kannan et al. [22] have reported three cases of OB in their three-year evaluation of bone-related biopsies. Reconstruction with vascularized bone grafts is a preferred treatment for bone pathologies [23] and a thorough curettage is recommended to prevent relapse [24]. Our patient underwent complete curettage, considering the young age and localized clinical presentation. A 25% recurrence rate has been reported for OB [5] but our patient remains disease-free on follow-up. 

## Conclusions

OB must be distinguished from similar bone-forming lesions, as accurate diagnosis is crucial for effective treatment planning. Bone-forming lesions often share clinical, radiographic, and histopathological characteristics with OB, which can complicate diagnosis. The conclusions drawn from the case are well-supported by the clinical, radiographic, and histopathological findings, which align with the typical presentation of OB of the mandible. The report provides valuable learning points for clinicians, including the importance of distinguishing OB and ensuring appropriate treatment. It also highlights key diagnostic features, such as lesion size, pain characteristics, and histopathological markers, that can guide accurate diagnosis and management. This case enhances understanding of the clinical behavior and optimal surgical approach for mandibular OB, aiding in the management. Our patient remains disease-free on follow-up, but long-term monitoring is essential to avoid recurrence, as the risk of relapse, though low, still exists.
